# A unified necessary condition for dynamic bow snap-through with prestress tuning in electrostatically actuated bistable microbeams

**DOI:** 10.1007/s11071-026-12816-x

**Published:** 2026-07-15

**Authors:** Sagi Tal, Lior Medina

**Affiliations:** School of Mechanical Engineering, Faculty of Engineering, Tel Aviv University, 6997801, Tel Aviv, Israel

**Keywords:** Bistability, Bow actuation, Dynamic switching, Basin of attraction, Bouncing, MEMS/NEMS

## Abstract

A curved bistable microbeam, subjected to electrostatic loading from an electrode facing its concave side, may exhibit bow snap-through (*BST*) at nearly half the voltage ($\approx 54\%$) when compared to actuation from the convex side. Previously, it was shown that introducing prestress can enable *BST* where it is otherwise unattainable. The current work extends the *BST* condition to include dynamic actuation, thus establishing dynamic bow snap-through (*DBST*) as a viable and sustainable form of actuation, since it can reduce actuation by ≈ 10 to 16%. To enable analytical derivation while retaining generality across various loading scenarios, the analysis employs an undamped single-degree-of-freedom reduced-order model obtained via Galerkin’s decomposition. The resulting formulation yields a necessary condition for *DBST*, expressed in terms of beam geometry, prestress, and initial conditions, forming a unified condition. Subsequent analysis shows that suddenly applied actuations, combined with prestress, can enable *BST* over a wider range of parameters, whereas excessive kinetic energy and/or prestress can suppress *DBST*. The proposed condition provides practical design guideline for energy-efficient, low-voltage and non-volatile bistable devices.

## Introduction

Bistability, namely the coexistence of two stable states under the same load, has been a highly sought-after attribute that attracted the attention of many. From what has started as a mere fascination with the nonlinearity and equilibrium curve that possesses a hysteresis with stable and unstable branches [1, 2], has eventually found its way to micro-electro-mechanical systems (MEMS) due to various possible applications, but also due to the interaction of said nonlinear equilibrium curve with the nonlinear nature of electrostatic load, used in MEMS as the main driving force and means of actuation [3–9].

Indeed, the incorporation of microstructures that possess bistability, such as curved beams and plates, to MEMS has sparked a spike in interest following the realisation that bistable structures can lead to bistable devices, able to distinguish between two main stable states, effectively introducing binary microstructures, along with a miscellany of applications [10]. Among such applications, it is possible to mention mechanical memories [11], micro-tweezers [12, 13], gas sensors [14], frequency-based logical gates [15], mass sensors [16], pressure sensors [17], flow velocity sensors [18], and much more. And while the applicative side of bistable structures was studied, the fundamental mechanics of electrostatically actuated bistable structures continued, with the purpose of seeking to uncover various phenomena that are only present when bistable structures are introduced to nonlinear, displacement-dependent loading. From the condition for electrostatic bistability [4], to the introduction of bifurcations [6], to the condition for symmetry breaking [19], and the effect of prestress [20, 21], to dynamic bistability [22], curved electrodes [23, 24] and the usage of multiple electrodes [25]. Among them, it is possible to point out one of the most impactful effects, which was always in the background one way or the other, but was largely taken for granted, until it was rigorously studied and scrutinised, namely the latching phenomena [26]. At its most basic definition, latching occurs when a zero-point load presents itself at the second stable branch [26–28]. Its presence, therefore, allows a bistable structure to hold itself at its second stable state under zero load, and as such, presents non-volatility. Such a property cannot be underestimated, since its very presence can promote bistable/binary and non-volatile structures and therefore prompt non-volatile devices, such as non-volatile mechanical memories (NVMM) [29, 30], mechanical encoders [31], non-volatile bistable mechanological metamaterials [32], non-volatile sensors that can be integrated into robots to enable edge computation [33], and non-volatile RF switches [34]. The incorporation of bistability with latching can therefore create devices that have the ability to save a result or an outcome after it was obtained, enabling a MEMS device to act both as a sensor and as a memory. Even more so, the act of “saving” a result in the structure/device/mechanical sensor is carried out without added voltage or power, making it power-efficient on the one hand, but also less prone to loss of memory due to outside interference such as radiation, present in inhospitable environments such as outer space [30].

Another property that has been studied over the past few years is the effect of added prestress. On the one hand, its presence has been deemed as a by-product of the fabrication process that needs to be taken into account in the modelling stage. The main reason was to account for its presence, since it affects experimental results, which adds a discrepancy with respect to stress-free models [27, 35–37]. Upon its incorporation, studies then sought not only to understand its effect on various phenomena present in bistable structures, but to harness it so that it would be possible to tune and effectively reprogram a bistable structure in real-time. From enabling bistability [21, 38–40], as well as latching [26, 41], to allow for frequency tuning [42], or to increase sensitivity [43].

As much as these properties are necessary, depending on the intended device, bistable microstructures “suffer” from high actuation voltage, prompting snap-through voltages in the range of $\approx \! 40 \, - \, 70$ V [4, 35, 44]. A range which makes the application of bistable devices problematic at best and less energy-efficient. This is true even when latching is present, since a device needs to first shift its equilibrium before the structure can hold itself and act as a memory. To remedy this problem, a new form of actuation was introduced in [45], where a bistable curved beam is actuated via an electrode facing the concave side of the beam, rather than from the more classical position, when the electrode is facing the convex side of the beam. In such a scenario, the beam is pulled away from its intended heading to induce accumulation of strain energy. At the end of the preloading, the beam is then suddenly released from the hold of the electrostatic load, causing the discharge of said strain energy in the form of kinetic energy, and if enough energy has been stored in the beam, then it will catapult and overshoot to the second stable state via what has been labelled as “bow snap-through” (*BST*). The study has not only shown that it is possible to induce a snap-through response, but that it is possible to do so with lower voltages, of about $\approx \! 54 \%$ when compared to convex facing electrodes. Later, a condition for the appearance of *BST* and dynamic *BST* (i.e., *DBST*) was formulated and extracted in [46], where the latter is induced in the presence of dynamic loads. However, it was evident that the resulting condition lies in a narrow space of required initial conditions and beam geometry. To circumvent that, prestress was introduced in [47], where it was shown that compressing a beam will enlarge the above space to accommodate additional initial conditions, as well as beam geometry. The main conclusion was that it is possible to tune a beam so that it will be more favourable to this form of actuation. However, it was also shown that there is an upper bound, since if the prestress is too large, then the permissible space will start to shrink due to the decrease in pull-in (*PI*) voltage values, along with the rise of the beam and increasing proximity to the electrode. As foretelling as it was, *DBST* was still largely unaddressed and ignored when prestress is present or introduced intentionally. The leading hypothesis here is that the same conclusion made for static *BST* can be made applicable to *DBST*, meaning that a beam can be tuned post-fabrication to make it more favourable to dynamic loads as well. Another hypothesis that justifies further exploration of *DBST* is that dynamic actuations from a concave-facing electrode will facilitate yet another decrease in voltage, similar to dynamic actuations emanating from a convex-facing electrode [48]. Since in the presence of dynamic loads, a snap-through can be carried out with a voltage that is reduced by about ≈10 to 15%. If such figures are apparent in bow activation, then that means that dynamic bow actuation can decrease the snap-through voltage by an additional percentage, making it a far more efficient and sustainable form of actuation on the one hand, while also enabling faster, dynamic actuations on the other hand. Such a realisation will go a long way in securing electrostatically bistable structures in MEMS in general, and the use of bow actuation, in particular.

To enable *DBST* and promote it as a viable form of actuation, we aim in the present study to combine dynamic actuation with prestress tuning so as to understand the impact of the latter on dynamic bow actuation, and characterise it via a case study. The study is completed with the formulation of a general and unified necessary condition for the appearance of *DBST* that takes into account possible initial conditions in the form of location and velocity, granted to the beam, all while in the presence of pretress.

## Model

**Fig. 1:**
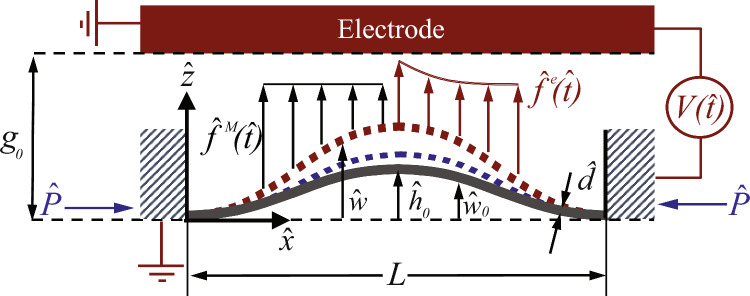
Model of an initially curved double clamped beam of length *L*, thickness $\hat{d}$, elevation $\hat{h}_0$, and initial configurations $\hat{w}_0$, with a maximum curvature $h_0$. The beam is prestressed by an axial force $\hat{P}$, causing it to shift its initial equilibrium, in grey, to the dashed blue line. The beam is actuated by an electrostatic load, originating from an electrode located at a distance of $g_0$ from the concave side of the beam (i.e., the “gap”). Dashed red line, $\hat{w}$, depicts the deformed equilibrium configuration caused by a distributed displacement-independent mechanical ($\hat{f}^M$, in black) loading or by displacement-dependent electrostatic load ($\hat{f}^e$, in red) resulting from a voltage difference *V*, either of which causes the beam to deflect to $\hat{w}$. Directions of the deflections and the direction of the loading are presented in their positive direction

Consider a flexible and curved double-clamped prismatic microbeam of length *L* and a rectangular cross-section, defined by its width $\hat{b}$ & thickness $\hat{d}$, as shown in Fig. 1. The beam is made of a homogeneous, isotropic and linearly elastic material, defined by its Young’s modulus *E* and Poisson’s ratio ν. Since the width $\hat{b}$ of the beam is larger than its thickness $\hat{d}$, an effective (plain-strain) modulus of elasticity $\tilde{E} = E/ \left( 1 - \nu ^2 \right)$ is used. The initial, as designed, shape of the beam is described by the function $\hat{w}_0 (\hat{x}) = \hat{h}_0 z_0 (\hat{x})$, where $\hat{h}_0$ and $z_0(\hat{x})$ are the initial elevation of the beam (located at its centre and taken with respect to the ends/boundaries of the beam), and is a non-dimensional function defined such that $\max \limits _{\hat{x} \in \left[ 0,L \right] } \left[ z_0 \left( \hat{x} \right) \right] = 1$, respectively. The beam is prestressed by an axial load $\hat{P}$, bearing on the edges of the beam, causing it to deform from its initial, as designed curvature, $\hat{h}_0$ (in grey), to a curvature with a maximum elevation *h* (in dashed blue) [21]. If the beam experiences a compressive axial force ($P/P_E > 0$), the resulting elevation will be higher than the stress-free elevation (i.e., $h > h_0$). Naturally, for a tensile axial force ($P/P_E < 0$), the beam will have a decreased initial elevation (i.e., $h < h_0$) [21]. Actuation of the beam is carried out via a distributed mechanical or electrostatic loads, $\hat{f}^m$ or $\hat{f}^e$, respectively, with the latter emanating from an electrode facing the concave side of the beam, located at a distance $g_0$ (termed henceforth as the “gap”), measured from the edges of the beam.

**Table 1:** Definitions of non-dimensional parameters

Parameter definition	Description
$x \triangleq \hat{x} / L$	Coordinate
$t \triangleq \hat{t} \sqrt{\tilde{E} I_{yy}/\left( \rho A L^4 \right) }$	Time
$w \triangleq \hat{w} / g_0, \; w_0 \triangleq \hat{w}_0 / g_0$	Elevation/initial elevation
$h_0 \triangleq \hat{h}_0/ g_0$	Initial midpoint elevation
$h \triangleq \hat{h} / g_0$	Midpoint elevation in post axial load application
$d \triangleq \hat{d}/g_0$	Thickness
$\alpha \triangleq \left( g_0^2 A\right) /\left( 2I_{yy}\right)$	Stretching parameter
$P \triangleq \left( \hat{P} L^2\right) /\left( EI_{yy}\right)$	Axial load
$c \triangleq \hat{c} L^2 / \sqrt{\rho A \tilde{E} I_{yy}}$	Damping parameter
$\beta ^M \triangleq \left( f^M L^4 \right) / \left( g_0 \tilde{E}I_{yy}\right)$	Mechanical loading parameter
$\beta \triangleq \left( \epsilon _0 \hat{b} V^2 \! L^4\right) /\left( 2 g_0^3 \tilde{E}I_{yy}\right)$	Voltage parameter

Taking into account that bow actuation can be carried out by either static or dynamic preloadings [45, 46], then combined with the above assumptions, the beam can be described by the following single non-dimensional integro-differential equation of motion [4, 7, 21, 49], per the non-dimensional definitions set out in Table 1

1 $$\begin{aligned}  &   \ddot{w} + c \dot{w} + w^{IV} - w_0^{IV}\nonumber \\  &   \quad + \left( P - \alpha \left( \int \limits _0^1 { \left( \left( w' \right) ^2 - \left( w'_0 \right) ^2 \right) dx} \right) \right) w'' - f \left( t \right) = 0 \end{aligned}$$

where the last term represents the applied distributed load, which can be electrostatic ($f^e$) or mechanical ($f^M$), described by

2 $$\begin{aligned} f^e \left( t \right) = \frac{\beta \left( t \right) }{\left( 1 - w \left( t \right) \right) ^2} \qquad f^M = - \beta ^M \left( t \right) \end{aligned}$$

Also, $$\hat{c}$$, $$I_{yy}$$ and ρ represent the dimensional viscous damping coefficient, second moment of area, and the density of the beam, respectively; $$\epsilon _0 \approx \! 8.854 \times 10^{-12} \;$$F/m accounts for the permittivity of vacuum, and *V* is the voltage difference between the beam and the electrode. Note that by setting the axial force to P=0, the current prestressed model formulation is reduced to its stress-free counterpart, analysed in [45, 46]. In addition, ${\left(\;\right)}^{\prime }$ and $$( \, )^{IV}$$ denote first and forth derivatives with respect to the non-dimensional spatial coordinate $$0 \le x \le 1$$ (i.e, $$\partial w/ \partial x$$ and $$\partial ^4 w/ \partial x^4$$, respectively), while $$\dot{ ( \, ) }$$ denotes derivative with respect to non-dimensional time *t* (i.e., $$\partial w/ \partial t$$).

Completing Eq. (1) are the following boundary conditions for a double clamped beam

3 $$\begin{aligned} w \left( 0, \, t \right) = w \left( 1, \, t \right) = 0 \qquad \frac{\partial w}{\partial x} \bigg |_{x \, = \, 0} = \frac{\partial w}{\partial x} \bigg |_{x \, = \, 1} = 0 \end{aligned}$$

with prescribed initial conditions $w_0 \left( x, \, 0 \right)$, $\dot{w}_0 \left( x, \, 0 \right)$, determined by the preloading scenario, which can be static or dynamic. Note that in contrast to the former study [47], the current analysis focuses on the latter, which in turn produces non-zero initial conditions, particularly non-zero initial velocity (i.e., $\dot{w}_0 \left( x, \, 0 \right) \ne 0$).

### Reduced order model

To allow for a rigorous study and subsequent extraction of a condition for dynamic bow snap-through (*DBST*) in the presence of prestress, a reduced-order (RO) model is constructed from Eq. (1) via Galerkin’s decomposition. Symmetry breaking, which can be manifested in a curved beam, depending on its geometry (i.e., *d* and $h_0$) and *P* values, can hamper latching [21, 26], and as such, hinder the ability of a bistable beam from converging to its second stable state in the absence of an external load. However, it is possible to circumvent that difficulty by using a double curved beam structure, limiting the response of the structure to its symmetric path from the outset, thereby avoiding the asymmetric path altogether. It is important to note, however, that the double curved beam structure should be composed of two perfectly identical beams that share the same geometry, as well as the same prestress value [38, 39, 50, 51]. Doing so will keep its limit points identical in terms of location to what a single beam will achieve, while increasing the voltage/load required to actuate the structure due to increased stiffness [51–53]. And although incorporation of several modes (i.e., more than one) will result in the formation of swerving unstable branches in a given equilibrium curve, the transition to bistability, and the snap-through response in particular, are determined by the first mode. As such, augmenting an RO model with additional modes will not provide new qualitative information beyond the decrease in voltage/load of a given equilibrium curve in general and of the limit points in particular, at least as far as symmetric behaviour is concerned [4, 35, 54]. These observations extend to dynamic snap-through as well, where it was shown that single-mode RO models can be of value when trying to understand the underlying dynamics in a given system, while extracting valuable observations from a seemingly simple model [7, 48, 55, 56]. Indeed, observations made using the single degree-of-freedom (DoF) RO model were found to be true in phenomena like dynamic trapping [57] and dynamic release [58], both of which have been experimentally validated in [41, 59]. For these reasons, it is possible to limit the model at hand to a single DoF, thus keeping the analysis simple while attaining critical observations. Following on that logic, the deformed (*w*) and initial shapes ($w_0$) of the beam are represented using a single mode/DoF approximation, provided by

4 $$\begin{aligned} w \left( x, t \right) \approx q \left( t \right) \varphi \left( x \right) \qquad w_0 \left( x \right) = h_0 \varphi \left( x \right) \end{aligned}$$

where $\varphi \left( x \right)$ corresponds to the first buckling eigenmode of a straight double-clamped beam.

Using the above expressions, Eq. (1) is converted to a single non-linear and ordinary differential equation in terms of the generalised, and time-dependent, coordinate *q*(*t*) (see [21] for further details), resulting in

5 $$\begin{aligned}  &   \ddot{q} + 2 \xi \omega _n \dot{q} + \omega _0^2 \left( q - h_0 \right) - P_E \left( \frac{P}{P_E} - \alpha \omega _0^2 \left( q^2 - h_0^2 \right) \right) \nonumber \\  &   \quad s_{11} q - f \left( t \right) = 0 \end{aligned}$$

while the corresponding single DoF representation of the loads, derived from Eq. (2), are

6 $$\begin{aligned} f^e \left( t \right) = \frac{\beta \left( t \right) }{2 m_{11} \sqrt{\left( 1 - q \left( t \right) \right) ^3}} \qquad f^M \left( t \right) = - \frac{\beta ^M \left( t \right) }{2 m_{11}} \end{aligned}$$

where the former is targeted towards the direction of the electrode (i.e., upwards, and as such, positive) and the latter is directed downwards, away from the electrode (and hence, negative). Here, $m_{11} = 3/8$ and $s_{11} = \pi ^2/2$ are the mass and stretching parameters of the beam, respectively, while $P_E$ is the non-dimensional Euler buckling load for a double-clamped beam, which can be taken as the ratio between the bending and stretchering parameters, with the former given by $b_{11} = 2 \pi ^4$, prompting $P_E \triangleq b_{11}/s_{11} = 4 \pi ^2$ [4, 21]. Also, $\omega _0 \triangleq \sqrt{b_{11}/m_{11}} = 4 \pi ^2/\sqrt{3}$, represents the linearised natural frequency of a straight stress-free beam (i.e., granted for $P = 0, \, h_0 = 0$).

The static counterpart of Eq. (5) can be found by multiplying it by $m_{11}$ and setting the inertial terms to nought (i.e., $\ddot{q} = 0, \, \dot{q} = 0$). Doing so will prompt the following static equation (matching the RO model in [21] for $q_2 = 0$)

7 $$\begin{aligned} b_{11} \left( q - h_0 \right) - \left( P - \alpha \left( q^2 - h_0^2 \right) s_{11} \right) s_{11} q - f = 0 \end{aligned}$$

where the corresponding loads are

8 $$\begin{aligned} f^e = \frac{\beta }{2 \sqrt{\left( 1 - q\right) ^3}} \qquad { {f^M = - \frac{\beta ^M}{2}}} \end{aligned}$$

As mentioned before, the presence of prestress changes the initial location of the beam, from $h_0$ to *h*. It is therefore paramount to isolate the initial location of the beam, *h*, from which the static response effectively begins. To do so, Eq. (7) is taken with zero load, f=0, granting the following implicit equation, cubic in terms of *q*

9 $$\begin{aligned} b_{11} \left( q - h_0 \right) - \left( P - \alpha \left( q^2 - h_0^2 \right) s_{11} \right) s_{11} q = 0 \end{aligned}$$

connecting $h_0$, the cut-section profile (via α), and the prestress *P*. Of the three roots, only two roots are of importance, namely the initial location of an equilibrium curve $q_I = h$, and the latching point $q_L$. The third solution represents a non-stable zero-point load, which does not play a part in this study and is therefore discarded [26].

To complete the formulation, the following damping ratio (ξ) and linearised natural frequency ($\omega _n$), present in Eq. (5), are derived for a curved and prestressed beam

10 $$\begin{aligned}  &   \xi = \frac{c}{4\pi ^2} \sqrt{\frac{3 d^2 P_E}{4d^2 \left( P_E - P \right) + 3P_E \left( 3 h^2 - h_0^2\right) }} \nonumber \\    &   \omega _n =\omega _0 \sqrt{1 + \frac{3}{4}\left( \frac{3 h^2 - h^2_0}{d^2}\right) - \frac{P}{P_E}} \end{aligned}$$

that include both *P* and *h* (see [47] for additional details). Note that by setting P=0 and $h_0 = h$, the damping and natural frequency will revert to their stress-free expressions [46].

The formulation of the RO model is completed by initial conditions, representing prescribed initial location $q \left( 0 \right) \triangleq q_0$ and velocity $\dot{q} \left( 0 \right) \triangleq \dot{q}_0$. In the current scenario, the preloading of the beam is carried out via a suddenly applied load, such as a finite pulse or ramp, depicting situations or scenarios where the beam achieves both a location and a non-zero velocity (i.e., $\dot{q}_0 \ne 0$), granting both strain and kinetic energies to the beam. This is in contrast to [47], where the beam was quasi-statically loaded to a prescribed location along its electrostatic equilibrium and held there until the load was dropped, causing the beam to accumulate only strain energy with zero velocity (i.e., $q_0 > 0, \, \dot{q}_0 = 0$). In such a scenario, the beam is released from the load that holds it, causing the accumulated strain energy to be released dynamically. For cases of non-zero damping, Eq. (5) is solved numerically via the Runge–Kutta-Fehlberg method, implemented by the dsolve function in MAPLE.

In the following sections, bow-actuation is studied in the presence of latching to allow the beam to converge to its second stable state in the presence of suddenly applied loads [26]. The analysis begins from a case study in the presence of damping, expressed using the *Q*-factor (where $Q \triangleq 1/\left( 2 \xi \right)$), showcasing possible responses achievable depending on the type of loading sequence, its intensity, and duration, all while the beam possesses various prescribed axial prestress values $P/P_E$. Following the case study, a general condition is then formulated using Eq. (5), to provide insights and upper limits for *DBST*.

## Case study

**Fig. 2:**
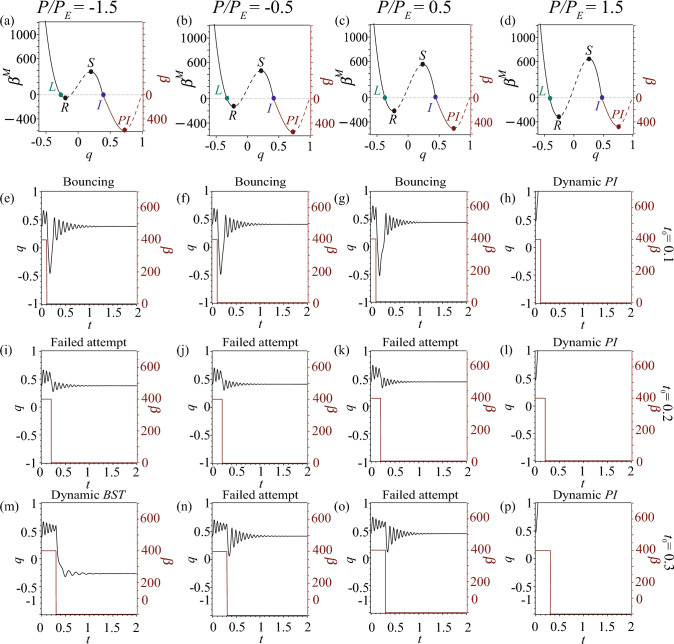
Case study of a beam with thickness $d \! \approx \! 0.143$ and initial elevation $h_0 \! \approx \! 0.428$, prestressed to four different values, corresponding to the parameters in Table 2. **a**–**d** Equilibrium curves, depicting the beam under mechanical ($\beta ^M$) and electrostatic loadings (β) in black and red, respectively, solved via Eqs. (7), (6) for each prestress. *I*, *S*, *R*, *L*, and *PI* denote the initial location, snap-through, release, latching, and pull-in points, respectively. Solid and dashed lines along the curves represent stable and unstable branches, respectively, while the grey dashed horizontal line corresponds to $\beta ^M = \beta = 0$. **e**–**p** Time histories, calculated for Q=10, and solved using Eq. (5) for a pulse signal, given by Eq. (11), with identical $\beta _{max} = 400$ and various pulse durations $t_0$

The analysis begins with a dynamic case study, where a beam, defined via its as-designed elevation $h_0 \! \approx \! 0.428$ and thickness $d \! \approx \! 0.143$, is introduced to four different prestress values, $P/P_E = -1.5, \, -0.5, \, 0.5, \, 1.5$, resulting in the four equilibrium curves given in Fig. 2a–d. Per the explanation above, each beam possesses a different *h*, but also a different latching point $q_L$, found via Eq. (9), as well as a different frequency $\omega _n$, calculated via Eq. (10). For the sake of clarity, these parameters are summarised in Table 2 for all four cases, along with their pull-in voltages $\beta _{PI}$, extracted from the plots, as well as their *BST* voltage, $\beta _{BST}$. The latter is calculated using the static *BST*condition from [47], granting a location $q_{BST}$, which is then substituted into the static equilibrium equation, namely Eq. (7). Note that similarly to [45–47], bistability is incurred only through the mechanical part of the equilibrium curve, since the purpose of the electrostatic load is to provide initial conditions (i.e., $q_0$ & $\dot{q}_0$) that will prompt a dynamic snap-through response (with convergence to the latching point), depending on the amount of total energy imbued to the beam during preloading. That being the case, the criteria for mechanical bistability, as well as for latching, are necessary conditions that must be met from the outset.

**Table 2:** Case study values for a beam with $d \! \approx \! 0.143$ and $h_0 \! \approx \! 0.428.$

$P/P_E$	*h*	$q_L$	$q_{PI}$	$\beta _{PI}$	$\beta _{BST}$	$\omega _n$
-1.5	$\approx \! 0.382$	$\approx \! -0.269$	$\approx \! 0.714$	$\approx \! 590.81$	$\approx \! 428.28$	$\approx \! 78.503$
-0.5	$\approx \! 0.413$	$\approx \! -0.327$	$\approx \! 0.723$	$\approx \! 508.72$	$\approx \! 441.2$	$\approx \! 84.083$
0.5	$\approx \! 0.443$	$\approx \! -0.373$	$\approx \! 0.732$	$\approx \! 470.02$	$\approx \! 445.49$	$\approx \! 89.449$
1.5	$\approx \! 0.471$	$\approx \! -0.411$	$\approx \! 0.741$	$\approx \! 470.02$	$\approx \! 439.67$	$\approx \! 94.607$

As was noticed in the analysis of the stress-free and prestressed beam models [46], the prescribed initial conditions play a key role in determining how the beam reacts. Generally speaking, we differentiate between four possible responses: An intra-well response, where the beam is given initial conditions that keep it in its first stable branch; an inter-well response, where the beam converges to the latching point; an inter-well response where the beam circles the fixed point for β=0 on the second stable branch (i.e., the latching point *L*), but ultimately converges to its initial location (a.k.a., “bouncing”), and a dynamic pull-in (*DPI*) response [26, 60]. To produce different initial conditions at the end of the dynamic preloading stage, we applied two types of suddenly applied loads, similar to what was done in [46]. Namely, a finite pulse, given by

11 $$\begin{aligned} \beta \left( t \right) = \beta _{max} \left( H \left( t \right) - H \left( t - t_0 \right) \right) \end{aligned}$$

and a finite ramp, given by

12 $$\begin{aligned} \beta \left( t \right) = \frac{\beta _{max}}{t_0} \left( 1 - H \left( t - t_0 \right) \right) t \end{aligned}$$

where *H*, $t_0$, and $\beta _{max}$ are the Heaviside function, the duration of the signals/loads, and the maximum voltage given to the beam, respectively.

Beginning with the first of the two signals, we load the beams via Eq. (11) with three different $t_0$ and identical $\beta _{max}$, to produce the responses given in Fig. 2e–p. The results show that it is possible to attain all four responses, depending on the prestress value and the duration of the pulse. It is also possible to note that the beam will achieve bouncing when possessing a certain prestress value, while the same beam will undergo a *DPI* or a *DBST* when under a different prestress. As such, it is possible to conclude that a successful transition to the second stable state via a dynamic load depends not only on the signal, which provides initial conditions when the load is dropped, but also on the prestress.

**Fig. 3:**
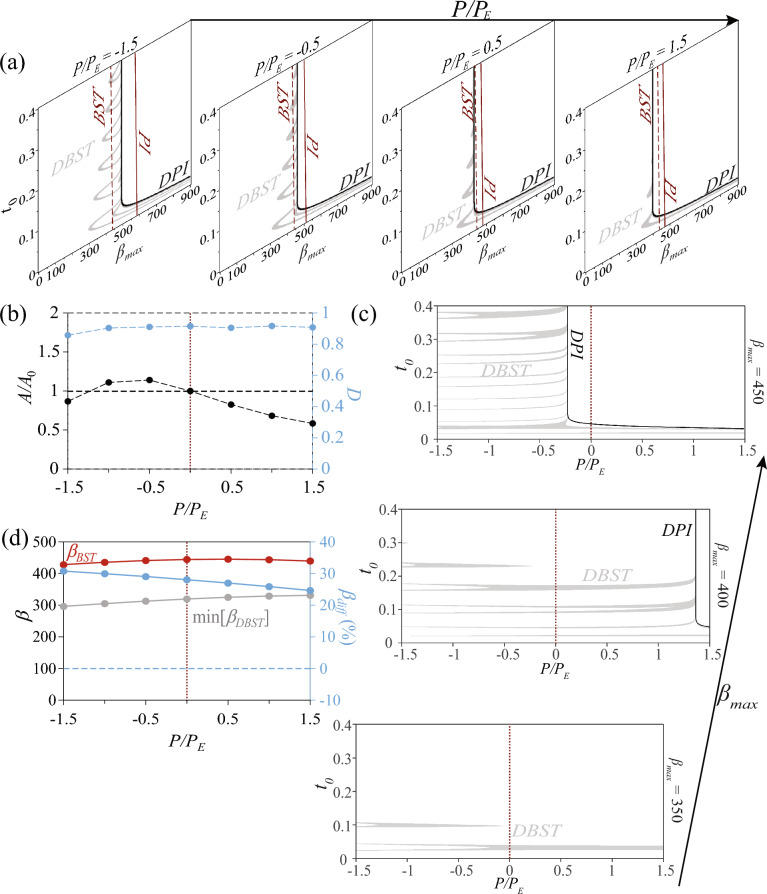
Actuation maps of a curved beam with thickness of $d \! \approx \! 0.143$ and initial elevation $h_0 \! \approx \! 0.428$ in the presence of an ambient damping defined by Q=10 and a pulse actuation signal, given by Eq. (11), corresponding to the case study from Fig. 2. Grey zones represent dynamic bow snap-through (*DBST*), while white regions below the dynamic pull-in (*DPI*) line (in black) represent bouncing. Also, dashed and solid red signify the static $\beta _{BST}$ and $\beta _{PI}$ voltages, respectively. **a** Maps for four $P/P_E$ values and **b** their corresponding characterisation, showing the ratio of *DBST* area against a stress-free beam ($A/A_0$) as a function of the prestress in black and the box counting dimension (*D*) in blue. Dashed black line represents $A/A_0 = 1$, while the vertical dotted red line signifies $P/P_E = 0$; **c** Actuation maps for three $\beta _{max}$ values, completing the maps from (**a**). **d** The decrease in voltage of the *BST* with respect to *DBST* in percentage (in blue), superimposed against $\beta _{BST}$ and the minimum $\beta _{DBST}$ as a function of $P/P_E$ shown in red and grey, respectively. A horizontal dashed blue line signifies zero difference in voltage

**Fig. 4:**
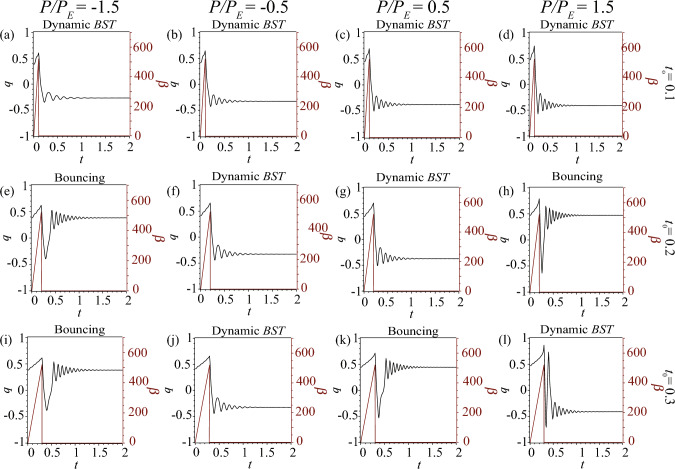
Case study of beams with identical thickness $d \! \approx \! 0.143$ and initial elevation $h_0 \! \approx \! 0.428$, prestressed to four different values, corresponding to the parameters in Table 2, and to the equilibrium curves from Fig. 2a–d. The time histories were calculated for Q=10, and solved using Eq. (5) for a ramp signal, given by Eq. (12), with identical $\beta _{max} = 520$ and various pulse durations $t_0$

To better witness the effect prestress has on *DBST*, we look at an aggregate of cases by constructing actuation maps on the $\{ \beta _{max}, \, t_0 \}$ plane, showing how the underlying parameters that define the pulse signal can change the response of the beam, for each $P/P_E$, while the environmental damping is set to Q=10. To construct such maps, Eq. (5) is solved for a multitude of cases via a sweep, conducted for a constant $t_0$ and varying $\beta _{max}$. If a $\{ \beta _{max}, \, t_0 \}$ combination produces a *DBST* response, achieving a location q(t)<0 at t=10, then it is marked on the map, and the calculation continues to the next $\beta _{max}$. If, however, a *DPI* is encountered during the sweep, then the sweep effectively ends, and the calculcation continues to the next $t_0$, to begin the next sweep. It is important to mention that there are two caveats to this calculation. First, the calculation needs to be fine enough to find areas in which *DBST* occurs, especially if one wants to quantify the *DBST* area (as was carried out in [47]). To achieve such a calculation with a reasonable degree of fidelity, we used increments of $\Delta t_0 = 2.5 \times 10^{-5}$ and $\Delta \beta _{max} = 0.125$. The second caveat is the running time, which is circumvented by exploiting the CPU multi-core, allowing for parallelisation of the computations. The result of the above procedure is given in Fig. 3a, showing how the *DBST* area, marked in grey, changes as $P/P_E$ rises from $P/P_E = -1.5$ to $P/P_E = 1.5$. To gain some perspective on the resulting maps, we superimposed $\beta _{BST}$ and $\beta _{PI}$, shown as dashed and solid red lines, for each prestress scenario. From the maps, it is possible to deduce that the *DPI* line, marked in black, is located below the *PI* voltage (i.e., $\beta _{DPI} < \beta _{PI}$), while also shifting to lower $\beta _{max}$ as $P/P_E$ increases. Both are to be expected. For the former, a dynamic pull-in response will always occur earlier than the static one, due to the added kinetic energy [7, 48]. For the latter, *h* increases with rising $P/P_E$, causing the beam to achieve closer proximity to the electrode, thereby limiting the allowable area for actuation. To better quantify the maps with rising $P/P_E$, we conducted both area (*A*) and box-counting dimension (*D*) calculations by counting boxes of varying sizes of $\left( 1/2 \right) ^i$, where i=10..15, allowing us to witness convergence for the calculation of *A* while also attaining *D* in the process. The result of both is in Fig. 3b, showing that the area, given with respect to the area of a stress-free beam, $A_0$, shown as the black curve, does indeed rise until $P/P_E = -0.5$, and then decreases for the remainder of the calculated range. In contrast to the area, the box-counting dimension does not change significantly. That is attributed to the overall shape of the *DBST* area, which keeps its nature and does not produce added erosion [7, 57, 60]. It is important to note that the range of this numerical calculation is limited to $0 \le t_0 \le 0.4$ for the sake of simplicity. If the calculation was to continue beyond it, then it is safe to assume that there is a maximum $t_0$ that would have been encountered, which decreases with rising $P/P_E$, as visible in the Fig. 3. To complete the analysis, we also produced actuation maps for the orthogonal plane, namely $\{ P/P_E, \, t_0 \}$ with increments of $\Delta \left( P/P_E \right) = 10^{-4}$ and $\Delta t_0 = 10^{-4}$ for three different $\beta _{max}$ values, producing the three maps in Fig. 3c. From this vantage point, it is possible to see how even a single DoF model can produce a complex response, showing how the *DPI* curve shifts to lower $P/P_E$ values as $\beta _{max}$ is increased on the one hand, while also showing how *DBST* areas emerge and vanish as a function of $P/P_E$, on the other hand. In addition, it is possible to discern from Fig. 3a how a small $t_0$ will necessitate large $\beta _{max}$ values, while also noticing that increasing $t_0$ beyond a certain point will not achieve *DBST*. On the other hand, compressed beams will allow a small $t_0$ while larger ones will not. Another interesting observation can be made by looking at the minimum *DBST* voltage ($\min \left[ \beta _{DBST} \right]$) and how its value changes with respect to $\beta _{BST}$, in each prestress scenario. Extracting both will result in the plot given in Fig. 3d, showing how the two change as a function $P/P_E$, presenting how $\min \left[ \beta _{DBST} \right] < \beta _{BST}$. Calculating the difference with respect to the latter of the two (in percentage) will prompt $\beta _{diff}$ (in blue), calculated via $\beta _{diff} (\%)= \left( \beta _{BST} - \min \left[ \beta _{DBST} \right] \right) /\beta _{BST}$, showing how *DBST* can lower β by as much as $\approx \! 30.8 \%$. However, such a gain diminishes with rising $P/P_E$, lowering it to $\approx \! 24.66 \%$ at $P/P_E = 1.5$. Translating the non-dimensional voltage parameter to dimensional voltage via the definition of β from Table 1 and computing the gain in terms of voltage will result in the following expression

13 $$\begin{aligned} G = \frac{\sqrt{\beta _{BST}} - \sqrt{\min \left[ \beta _{DBST} \right] }}{\sqrt{\beta _{BST}}} \times 100 \end{aligned}$$

showing that voltage-wise, *DBST* can lower the voltage by $G\approx \! 13.201 \% - \! 16.813 \%$ in this loading scenario, and for the presented prestress range.

The case study above suggests two things. Firstly, prestress can influence the amount of possibilities for *DBST*, as was seen in the static case [47]. However, increasing it beyond a certain $P/P_E$ can have diminishing returns, since the *DPI* etches the resulting area with rising prestress. Secondly, *DBST* can help decrease the voltage necessary for a snap-through response with respect to static *BST*. However, this decrease in voltage is affected by the $P/P_E$, which can negate the effect as the prestress rises.

To better cement these understandings, whilst also aiming to witness what is consistent when taking a different form of actuation that can introduce different initial conditions, we conducted the same type of analysis on the ramp signal, Eq. (12), producing the time histories in Fig. 4. This case study shows again that not only do the responses depend on $\beta _{max}$, $t_0$ and $P/P_E$, as is in the pulse signal, but also how they differ. Realising that the amount of energy being input to the beam is different in each loading scenario is the key, as it affects the initial conditions given to the beam. Specifically, the amount of energy in this scenario is half of the previous signal, since the voltage is not constant but rises from β=0 to $\beta = \beta _{max}$ for the same duration $t_0$.

**Fig. 5:**
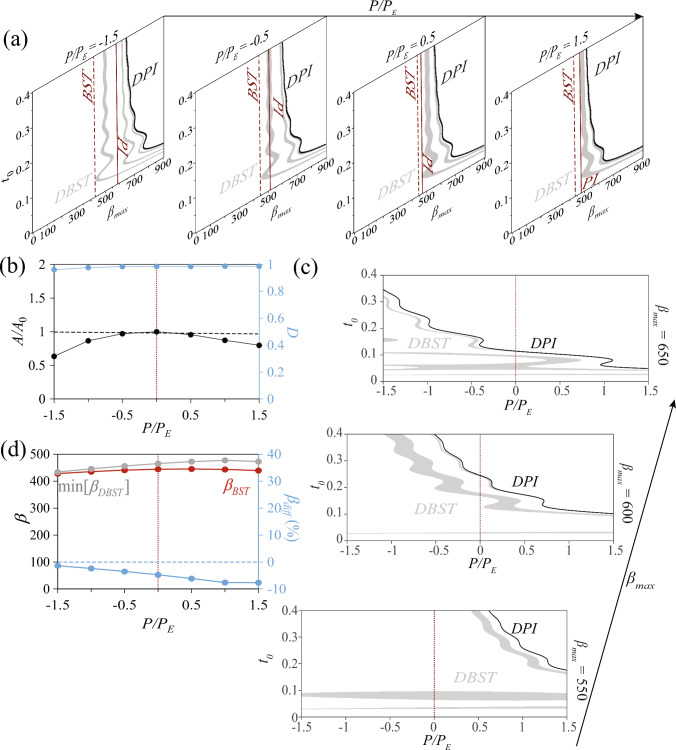
Actuation maps of a curved beam with thickness of $d \! \approx \! 0.143$ and initial elevation $h_0 \! \approx \! 0.428$ in the presence of an ambient damping defined by Q=10 and a ramp actuation signal, given by Eq. (12), corresponding to individual case studies from Fig. 4. Grey zones represent dynamic bow snap-through (*DBST*), while white regions below the dynamic pull-in (*DPI*) line (in black) represent bouncing. Also, dashed and solid red signify the static $\beta _{BST}$ and $\beta _{PI}$ voltages, respectively. **a** Maps for four $P/P_E$ values and **b** their corresponding characterisation, showing the ratio of *DBST* area against a stress-free beam ($A/A_0$) as a function of the prestress in black and the box counting dimension (*D*) in blue. Dashed black line represents $A/A_0 = 1$, while the dotted red line signifies $P/P_E = 0$; **c** Actuation maps for three $\beta _{max}$ values, completing the maps from (**a**). **d** The decrease in voltage of the *BST* with respect to *DBST* in percentage (in blue), superimposed against $\beta _{BST}$ and the minimum $\beta _{DBST}$ as a function of $P/P_E$ shown in red and grey, respectively. A horizontal dashed blue line signifies zero difference in voltage

**Fig. 6:**
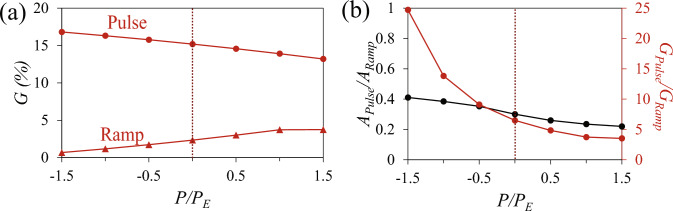
Summary of the case study, showing **a** the gain in voltage for the pulse and ramp signals, corresponding to Figs. 3d, 5d, respectively, calculated via Eq. (13); **b** The ratio between the *DBST* area of the pulse signal ($A_{Pulse}$) and the *DBST* area of the ramp signal ($A_{Ramp}$) in black, and the ratio between the voltage gain of the pulse signal ($G_{Pulse}$) and the voltage gain in the ramp signal ($G_{Ramp}$) red. The vertical dotted red line signifies $P/P_E = 0$

Conducting a sweep once more for the ramp signal will result in the actuation maps in Fig. 5, showing a similar trend to what was shown in [46] for the stress-free beam. More specifically the response of the beam levels off as $t_0$ increases, with the *DPI* curve and the *DBST* area getting closer to each other. Such a thing is to be expected since with rising $t_0$, the response of the beam will converge to a quasi-static response, where the strain energy is more dominant with respect to the kinetic part. Ergo, $\dot{q}_0$ is decreasing as $t_0$ is increased. Note that $\beta _{PI} < \beta _{DPI}$ in this scenario, all because the energy is smaller, allowing for a larger permissible actuation area, all while $\beta _{DPI}$ is getting smaller with rising $P/P_E$, as was in the pulse signal. Also note that in contrast to the pulse signal, $\beta _{BST}$ and $\min \left[ \beta _{DBST} \right]$ are much closer to each other, suggesting that in this loading scenario, the ramp does not provide a clear advantage when considering how much this signal can save voltage. Indeed, when calculating the gain in terms of voltage according to Eq. (13), it is evident that the “advantage” in this scenario and prestress range is minute, ranging between $G \approx \! 0.68\%$–3.75%. This is attributed again to the amount of energy introduced to the beam.

At this point, it is important to point out that while the above results are granted for an ambient damping defined by Q=10, decreasing the damping (or increasing *Q*) will cause the resulting patterns to become eroded while maintaining the overall behaviour, which will cause the box-counting dimension (*D*) to reduce in value. When considering *DBST*, pulse and ramp signals were computed for Q=10 and for Q=100 for a stress-free beam in [46], showing that the added erosion kept the overall shape of the actuation maps, while dynamic snap-through and pull-in voltage thresholds shifted to lower values, asymptotically reaching perfect vacuum (i.e., $Q \rightarrow \infty$). Indeed, the same behaviour was witnessed in other works as well, showing that a single DoF RO lumped model will show erosion in basin of attraction analysis, as well as actuation maps, all while keeping the overall qualitative shape and external boundaries of the maps [26, 55–58].

When observing the total behaviour of the beam under a ramp signal, it is possible to observe that the rise in *DBST* area peaks at $P/P_E = 0$ and not at $P/P_E = -0.5$, as was the case in the pulse signal. That tells us that it is possible to attain *DBST* at higher $P/P_E$ values when less energy is imparted to the beam. As such, it is possible to deduce that *DBST* will increase and decrease depending on the energy imparted to the beam, determined by the velocity given to the beam, as well as its prestress value. The combination of both is therefore paramount and is at the heart of the current analysis. At this point, it is important to note that the actuation maps here do not account for bouncing, but only for convergence to the latching point, thereby producing an image that while partial, is quite telling nonetheless.

To better understand the difference between the two signals and their effect on the beam, it is possible to visualise the trend in voltage gain, as well as the ratio between the *DBST* areas and the voltage gains themselves, as shown in Fig. 6. When looking at the voltage gain, Fig. 6a, it is clear that a pulse signal can provide a clear advantage and decrease the amount of voltage required to produce a *DBST* when comparing it to the ramp. That is attributed to the amount of energy imparted onto the beam in the former, which is twice as large compared to the latter. However, that is not without drawbacks, since the increased energy in the pulse can cause the beam to reach a dynamic pull-in response at a lower voltage compared to the ramp signal, thus introducing a trade-off. In Fig. 3, 5, we observed the amount of area that prompted *DBST* as a function of prestress compared to the *DBST* area of a stress-free beam. That analysis was instrumental in figuring out how the area increases and decreases in each scenario. However, it does not allow a clear comparison between the two loads, since both are compared to a stress-free beam under the same type of load. Figure 6b shows a comparison between the two signals by showing the ratio of the areas, as well as the voltage gain ratio as a function of prestress in black and red, respectively. The comparisons show that while each signal peaked at a different prestress, the ratio between the two shows a clear advantage to the ramp signal, with the ratio showing that $A_{Pulse} < A_{Ramp}$, since for that type of signal, more options are available for *DBST*. However, in terms of voltage, it is visible that $G_{Pulse} > G_{Ramp}$.

## Dynamic bow snap-through condition in the presence of prestress

Since the goal is to derive a general condition for all loading types, carried out in whatever signal/s, circumstances and/or sequences, ultimately granting the beam the necessary location and/or velocity to allow for *DBST*, the condition is based on prescribed initial conditions, $q_0, \dot{q}_0$. In so doing, we effectively replace the question “under what load and type of signal can one carry out a successful *DBST*?” with “what initial conditions should one reach to obtain *DBST*?” The result of the process would therefore be a general condition that provides a field of initial conditions at which *DBST* comes to pass, which can be applicable for all types of loads. By finding said field of initial conditions, all that remains for one to do is connect a specific/desired loading scenario to the location and velocity these provide at the end of the loading cycle. Furthermore, convergence to the latching point ultimately depends on the damping, as was seen in the preceding case study, since a beam can converge to the latching point or bounce back depending on the frequency of the beam, which can change due to the prestress, as well as the amount of energy provided to the beam by the load. Since the goal of the current study is to provide a simple enough condition to be used as a guide in the design of bistable beams actuated via bow actuation, we derive a condition for an undamped environment (i.e., for which ξ=0 or $Q \rightarrow \infty$), allowing analytical derivation. However, since the resulting condition is developed for $Q \rightarrow \infty$, it therefore provides a necessary condition that satisfies an inter-well response but may or may not achieve convergence to the latching point. Despite all the above, such a condition, as brought here, provides key insights as well as serves as a preliminary tool when intending on using bow-actuation when designing curved beams. Following the derivation, the condition is first examined for mechanical load, to better understand it on the one hand, as well as facilitate the methodology for its analysis, on the other hand.

Considering the above assumptions, then the solution of Eq. (5) is fully parametrised by its initial conditions $q_0, \, \dot{q}_0$ for zero load (f=0). Calculating the first integral of Eq. (5) will yield

14 $$\begin{aligned} \frac{m_{11} \dot{q}^2}{2} = H - U \left( q \right) \end{aligned}$$

where $U \left( q \right)$ and *H* are the potential energy and the Hamiltonian, respectively, written as

15 $$\begin{aligned}&U \left( q \right) = \frac{1}{2} b_{11} \left( 1 -\frac{P}{P_E} - \alpha \frac{s_{11}}{b_{11}} h_0^2 \right) q^2 + \frac{1}{4} \alpha s_{11}^2 q^4 - b_{11} h_0 q \end{aligned}$$

16 $$\begin{aligned}&H = \frac{m_{11} \dot{q}_0^2}{2} + U \left( q_0 \right) \end{aligned}$$

**Fig. 7:**
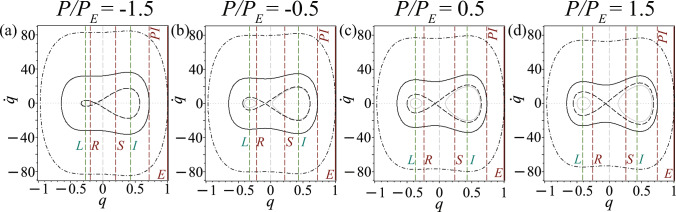
Phase plane trajectories in an undamped environment (ξ=0) of the beams from Fig. 2. Three main trajectories are depicted for each beam, representing the intra-well response in solid grey, the inter-well response in solid black, and the separatrix in dashed black, set between the last two. Black dash-dot black trajectory is obtained for $q_0 = 1$ and $\dot{q}_0 = 0$. *I*, *S*, *R*, *L*, and *PI* mark initial location, snap-through, release, latching and pull-in points, respectively. *E* represents the location of the electrode, $q_0 = 1$. Grey vertical dashed and horizontal dotted lines represent $q_0 = 0$ and $\dot{q}_0 = 0$, respectively

Further simplification of Eqs. (14)–(16) will result in the following expression, representing the phase plane [48]

17 $$\begin{aligned}  &   \dot{q}^2 - \dot{q}_0^2 - \omega _0^2 \left( \left( 1- \frac{P}{P_E} + \frac{\alpha s_{11}}{2 P_E} \left( q^2 + q_0^2 - 2h_0^2 \right) \right) \right. \nonumber \\  &   \left. \left( q + q_0 \right) - 2 h_0 \right) \left( q - q_0 \right) = 0 \end{aligned}$$

which can be used to further characterise the beams from Fig. 2. Substituting beam values from Table 2 in Eq. (17) and setting $\dot{q} = 0$ will prompt the undamped phase plane trajectories shown in Fig. 7. The two closed loops in grey are granted for $q_0 = 0.566, \, 0.6, \, 0.63, \, 0.66$ for $P/P_E = -1.5, \, -0.5, \, 0.5, \, 1.5$, respectively, showing two independent intra-well trajectories, one around each fixed point, corresponding to the initial location. The trajectories shown in black dashed lines represent the separatrix, and are granted for $q_0 \approx 0.626, \, 0.566, \, 0.606, \, 0.645, \, 0.682$, showing the boundary between intra- and inter-well trajectories. Furthermore, it is evident that as $P/P_E$ rises, the bigger the trajectory that can be carried out in a beam. Also note that with rising prestress, the required initial location to attain the sepratrix rises as well, setting the beam closer to $q_{PI}$, limiting the number of options a beam can have. However, that is true in this particular instance. In the aggregate, prestrerss can actually increase the amount of possibilities due to the presence of a second latching threshold, which forms when the prestress is high enough [26, 47]. These notions, while true for static *BST*, still hold true for *DBST* as well, since the former is a private case in the dynamic case. However, what sets *DBST* from *BST* apart is the fact that there can be a dynamic pull-in (*DPI*) response, which is determined by the type of signal bearing on the beam [48, 57], which the current formulation, while being general, assumes that the dynamic load only provides initial conditions to the beam. That being said, there is yet another boundary that is similar to a dynamic pull-in, the so-called “dynamic upper-bound” that sets the location of the electrode as the permissible boundary for any dynamics in this system. Calculating the trajectory for $q_0 = 1, \, \dot{q}_0 = 0$ will prompt the dash-dot lines in Fig. 7, acting as the most developed trajectory that can be introduced to the beam in the presence of an electrode. At this stage, it is important to note two things. The first is that this dynamic upper bound is relevant only for electrostatic load and bears no relevance for mechanical load. Second, while the phase plane trajectories depicted in Fig. 7 were plotted for $\dot{q} = 0$, they represent all possibilities and permutations of $q_0$ and $\dot{q}_0$ for *DBST*. The dynamic upper bound is shown later in the paper, when its relevance is required in the electrostatic condition.

To identify the separatrix, $\dot{q}$ is set to nought, disclosing where a phase plane trajectory crosses the $\dot{q} = 0$ axis. By doing so, the term $\dot{q}^2$ is eliminated, providing us with the following implicit equation

18 $$\begin{aligned}  &   \dot{q}_0^2 + \omega _0^2 \left( \left( 1- \frac{P}{P_E}+ \frac{\alpha s_{11}}{2 P_E} \left( q^2 + q_0^2 - 2h_0^2 \right) \right) \right. \nonumber \\  &   \quad \left. \left( q + q_0 \right) - 2 h_0 \right) \left( q - q_0 \right) = 0 \end{aligned}$$

which is quartic in terms of *q*. Note that deriving the separatrix for a zero initial velocity will result in an implicit cubic equation, multiplied by a trivial solution $q = q_0$ [47]. However, in the present case, that cannot be stated, and such a simplification cannot be made, all because there is a non-zero initial velocity present in the system. Since the condition is quartic, it has four different solutions, depending on the geometry, initial condition and prestress.

Looking at the possible solutions Eq. (18) can have, there can be several options, as depicted in Fig. 7. It either has two different roots (each with a multiplicity of two) when there is only one intra-well response (i.e., one around $q_I$ or one around $q_L$); four distinct roots, representing two independent intra-well responses, represented by the grey trajectories. Two roots, located at the outskirts of the phase plane (each with a multiplicity of two), representing an inter-well response, depicted by the black trajectories, and three roots (with one of them repeating itself), showcasing the transition from an intra-well to an inter-well response, represented by the separatrix in dashed black trajectories. To satisfy three roots, a vanishing discriminant is demanded, prompting the following implicit equation for a beam with a rectangular cross section (i.e., having $\alpha = 6/d^2$)

19 $$\begin{aligned}  &   6912 G^3 + 1152 P_E^2 F^2 G^2 + 5184 P_E^2 F G \frac{h_0^2}{d^2} \nonumber \\  &   \quad + 3888 \frac{P_E^2}{b_{11}^2} \frac{h_0^4}{d^4} + \frac{48}{s_{11}^4} \left( F G + s_{11} P_E \frac{h_0^2}{d^2} \right) F^3 = 0 \end{aligned}$$

where the functions *F* and *G* are expressed by

20 $$\begin{aligned} F&= b_{11} \left( 1 - \frac{P}{P_E} - 6 \frac{s_{11}}{P_E} \left( \frac{h_0}{d} \right) ^2 \right) \end{aligned}$$

21 $$\begin{aligned} G&= 3 s_{11}^2 \left( \frac{q_0}{d} \right) ^4 + \left( \frac{q_0}{d} \right) ^2 F - 2 b_{11} \frac{h_0}{d} \frac{q_0}{d} + m_{11} \left( \frac{\dot{q}_0}{d} \right) ^2 \end{aligned}$$

expressing the combination of geometry ($h_0, \, d$), initial conditions ($\dot{q}_0, \, q_0$) and prestress ($P/P_E$) required for a successful trajectory around $q_I$ and $q_L$, that may produce *DBST*. Note that the condition takes into account the presence of prestress, as well as initial velocity, possible only via dynamic actuation. As a result, the above condition extends the one from [46], where a dynamic actuation was taken into account, but prestress was left out, as well as the condition from [47], where prestress was included, but did not include dynamic actuation. That being the case, the expression analysed here unifies both end cases to a single condition that explores the combined contribution and impact of both parameters, thus making the condition in [46] a private case when $P/P_E = 0$ and the condition from [47] a private case when $\dot{q}_0 = 0$.

### Mechanical load

Before venturing into electrostatic load, we begin the study of Eq. (19) for displacement-independent mechanical load. The main distinction between the two loads is that the electrostatic load is displacement-dependent and, due to that, is upper-bounded by a *PI* response [9], while the mechanical load is displacement-independent and lacks such a boundary.

**Fig. 8:**
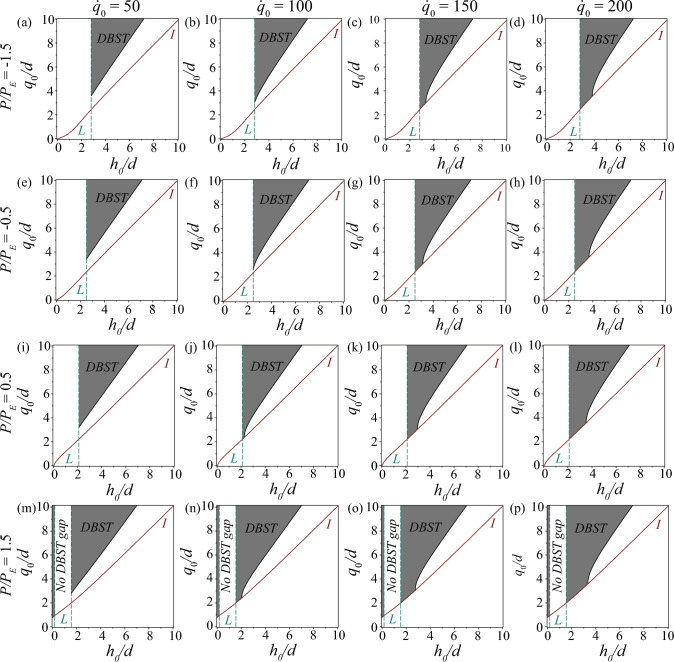
Condition for dynamic bow snap-through (*DBST*) in a mechanically loaded beams in terms of initial location ($q_0$), and under different combinations of prestress and initial velocity. Solid red and black lines correspond to the initial location (*I*) and the *DBST* necessary condition, respectively, while the dashed green line represents the latching condition, *L*. The dark grey areas represent where the conditions are met

Solving the condition, Eq. (19), for different prestress and initial velocities (i.e., $P/P_E, \, \dot{q}_0 = const$) will result in a condition for each combination, as given in Fig. 8 for four different $P/P_E$ and four different $\dot{q}_0$. Note that we show the condition in the $\{ h_0/d, q_0/d \}$ plane, similarly to how the mechanical condition was represented in [47]. Such a representation is quite prevalent for mechanically loaded structures [51, 53], which in turn simplifies the condition, as the ratios are already present in Eq. (19), but also eliminates $g_0$ altogether, since it does not have any bearing here. The resulting conditions in Fig. 8, marked in black, are granted by finding the roots of Eq. (19) for each $h_0/d$, located at the permissible range of q≥h, where $h = q_I$ is found from Eq. (9), marked via the red curve. In addition, since the latching condition is also necessary [26], then it is introduced as well, and is superimposed on the $\{ h_0/d, q_0/d \}$ plane as the vertical green dashed line. We recall that for $P/P_E < 1$, there will be only one latching threshold (or a single critical elevation, $h_{cr}$) from which latching is possible. At $P/P_E > 1$, another threshold appears ($h^{\left( 2 \right) }_{cr}$), serving as an upper bound for low $h_0/d$. At $P/P_E \ge 2$, both thresholds disappear, and latching is present for all $h_0/d$ [26, 47]. Adding the condition for *DBST* adds a lower bound, shown as the black curve, making each point above it a potential combination for *DBST*. As such, the area above the condition is marked in its entirety as the area at which *DBST* is made possible. At first glance, it appears that as the initial velocity is increased, so does the area, since the added kinetic energy extends the condition to include additional options, to be included at the same prestress, particularly in beams with higher $h_0/d$, not possible when slow quasi-static preloading is carried out. This trend is consistent with rising $P/P_E$, which occurs simultaneously with the decrease in the latching threshold. As we can see at $P/P_E = 1.5$, another latching boundary is forming at a low $h_0/d$, thus introducing additional possibilities for *DBST*, effectively increasing the permissible area.

**Fig. 9:**
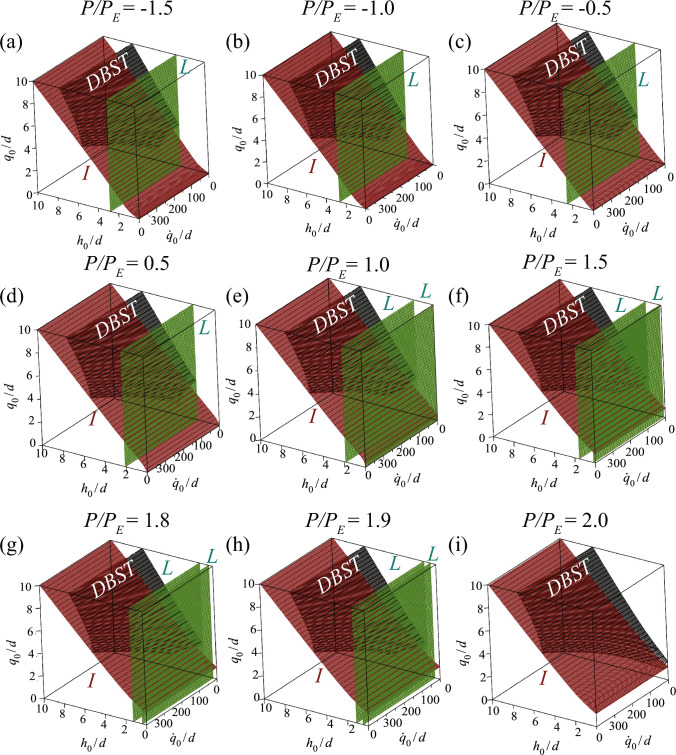
Three-dimensional representation of the dynamic bow snap-through (*DBST*) condition in a mechanically loaded beams in terms of initial location ($q_0$) and velocity ($\dot{q}_0$) under different constant prestress. Red, green and black surfaces correspond to the initial location (*I*) point, the latching location (*L*), and the *DBST* necessary condition

At this point, it is important to point out that in contrast to [47], the mechanical condition here depends on an additional parameter, namely $\dot{q}_0$. As such, if the mechanical condition depends solely on two parameters in [46], namely $h_0/d$ and $q_0/d$, and three parameters in [47], with the addition of $P/P_E$, then the present case introduces a mechanical condition in a four-dimensional space, effectively constructing a tesseract. To show a representation of the condition, we span the two-dimensional conditions from Fig. 8 in the space of $\{ h_0/d, \, q_0/d, \, \dot{q}_0/d, \}$ for different constant $P/P_E$. Doing so will span the tesseract via its three-dimensional projections, showing its evolution as a function of $P/P_E$, as shown in Fig. 9. The plots introduce three surfaces, namely the initial location (*I*), the latching thresholds (*L*) and the *DBST* surface in red, green and black, respectively, showing how they all evolve with rising prestress. The volume, trapped between the surfaces, represents the volume that allows for *DBST*. As the condition reaches $P/P_E = 1$, another surface appears at $h_0/d = 0$, as expected, and introduces a second volume. As $P/P_E$ continues to rise, the two latching surfaces get closer and closer, increasing the overall permissible volume, until the two thresholds meet at $P/P_E = 2$, providing us with a single volume.

To quantify the change of the permissible volume as $P/P_E$ increases, *DBST* curves, calculated in the $\{ h_0/d, \, q_0/d \}$ plane that span the three-dimensional projections, were fitted with a twelve-order polynomial using the Least Squares Method, producing an approximate function $q_0/d \! \approx \! \mathcal {F} \left( h_0/d \right)$, which is then used to calculate the area via direct integration. Multiplying each area by a $\Delta \left( \dot{q}_0/d \right)$ to attain a volume element $\Delta V_i$ and summing the contributions of all incremental volumes (or elements) will provide the overall trapped volume. If the condition intersects the latching threshold ($h_{cr}$), then the integration is carried out from the latching boundary to the maximum value taken in the plots, set at $q_0/d = 10$, according to

22 $$\begin{aligned}  &   \Delta V_{i_1}^{(1)} \left( \frac{P}{P_E}, \, \frac{\dot{q}_0}{d} \right) \nonumber \\  &   \quad = \Delta \left( \dot{q}_0/d \right) \left( \int \limits _{h_{cr}}^{ \left( h_0/d \right) _{max}} \left( \left( q_0/d \right) _{max} - \mathcal {F} \left( \frac{h_0}{d} \right) \right) \; d \left( h_0/d \right) \right) \end{aligned}$$

where $\left( h_0/d \right) _{max}$ represents the highest elevation-to-thickness ratio that corresponds to $\left( q_0/d \right) _{max}$. If, however, the condition does not intersect the latching threshold, but the initial location, due to the presence of a non-zero initial velocity, then the integration is split into two parts, according to

23 $$\begin{aligned}&\Delta V_{i_2}^{(1)} \left( \frac{P}{P_E}, \, \frac{\dot{q}_0}{d} \right) \nonumber \\&\quad = \Delta \left( \dot{q}_0/d \right) \left( \int \limits _{h_{cr}}^{ \left( h_0/d \right) _{min}} \left( \left( q_0/d \right) _{max} - q_I \left( \frac{h_0}{d} \right) \right) \; d \left( h_0/d \right) \right) \nonumber \\&\quad +\Delta \left( \dot{q}_0/d \right) \left( \int \limits _{\left( h_0/d \right) _{min}}^{ \left( h_0/d \right) _{max}} \left( \left( q_0/d \right) _{max} - \mathcal {F} \left( \frac{h_0}{d} \right) \right) \; d \left( h_0/d \right) \right) \end{aligned}$$

where $\left( h_0/d \right) _{min}$ signifies the minimum $h_0/d$ of the condition, representing the intersection with the initial location $q_I = q_I \left( h_0 / d \right)$. For $P/P_E > 1$, there is an additional volume, trapped between $0 \le h_0/d \le h_{cr}^{\left( 2 \right) }$, calculated via

24 $$\begin{aligned}  &   \Delta V_{i_3}^{(3)} \left( \frac{P}{P_E}, \, \frac{\dot{q}_0}{d} \right) \nonumber \\  &   \quad = \Delta \left( \dot{q}_0/d \right) \left( \int \limits _{0}^{ h_{cr}^{\left( 2 \right) } } \left( \left( q_0/d \right) _{max} - q_I \left( \frac{h_0}{d} \right) \right) \; d \left( h_0/d \right) \right) \end{aligned}$$

Summing the volumes of all elements over the entire range of $\dot{q}_0/d$ will result in the total *DBST* volume for each $P/P_E$ according to

25 $$\begin{aligned} V \left( \frac{P}{P_E} \right) \approx \sum _{j=1}^{3} \sum _{i_j=1}^{n_j} \Delta V_{i_j}^{(j)} \left( \frac{P}{P_E}, \, \frac{\dot{q}_0}{d} \right) \end{aligned}$$

where $n_j$ ($j = 1, \, 2, \, 3$) marks the number of elements for each case such that $i_1 = 1.. n_1, \, i_2 = 1..n_2, \, i_3 = 1..n_3$.

**Fig. 10:**
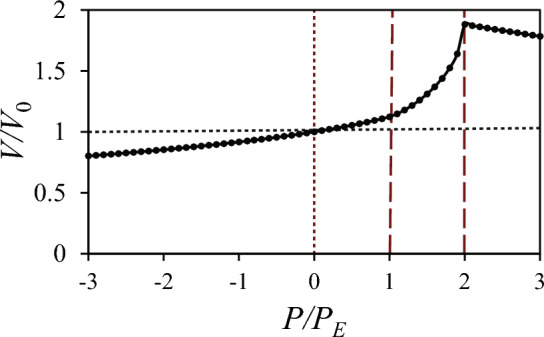
The volume *V* of the dynamic mechanical bow snap-through condition from Fig. 9 as a function of the prestress, $P/P_E$, with respect to the volume of a stress-free curved beam (i.e., $V_0$ is *V* for $P/P_E = 0$). The dotted horizontal black line represents $V/V_0 = 1$. Vertical dotted and dashed red lines represent $P/P_E = 0$ and $P/P_E = 1, \, 2$, respectively

Before conducting such a calculation, one must first find the thickness of the element $\Delta \left( \dot{q}_0/d \right)$. To find it, a convergence analysis was carried out until the total volume converged to a value with an error of less than 1% with respect to the finest mesh, showing that an increment of $\Delta \left( \dot{q}_0/d \right) = 5$ is sufficient for this calculation when comparing it to a mesh with elements defined by $\Delta \left( \dot{q}_0/d \right) = 2.5$, for all three scenarios. Conducting this calculation for each three-dimensional projection and extracting the volume trapped for each $P/P_E$ will result in the plot in Fig. 10, showing an increase with rising prestress. The rise is attributed to the latching condition, which shifts to lower $h_0/d$ values as $P/P_E$ rises, as well as to the appearance of a second latching threshold at $P/P_E > 1$, prompting a non-linear rise in volume, until $P/P_E = 2$, at which the volume will start to decrease. This means that when conducting a mechanical-based pull on the beam from its concave side, it is preferable to compress the beam, so as to attain a higher amount of possibilities in terms of initial conditions and geometry. However, increasing the compression beyond $P/P_E = 2$ will have a negating effect, since the *DBST* surface is being “overrun” from below by the initial location, which continues to increase with rising $P/P_E$, shrinking the overall *DBST* volume.

### Electrostatic load

Upon unravelling the behaviour of the beam and its ability to prompt a dynamic bow snap-through when under mechanical load, we now move on to incorporate electrostatic loading. Unlike the previous load, the dependence on the gap cannot be removed since the load is displacement-dependent, which in turn introduces a *PI* response [4, 6, 61]. To find the *PI* curve, one needs to substitute $f = f^e$ from Eq. (8) in Eq. (7), isolate β and differentiate the equilibrium equation according to dβ/dq, to find the extremum points. Since the extremum points in a single curved beam overlap with the limit points [4, 21, 62], then demanding dβ/dq=0 will result in the so-called limit point equation, cubic in terms of *q* (see [21] for additional details). Since the operational range of the electrostatic load is between the initial location and the pull-in point ($q_{PI}$), $h \le q \le q_{PI}$, then only one root, representing $q_{PI}$, is of significance. Isolating that root will prompt a function that depends on the geometry of the beam, as well as its prestress, $q_{PI} = q_{PI} \left( h_0, \, d, \, P/P_E \right)$. As was pointed out before, *DPI* depends on the type of load signal/scenario/sequence being used to actuate the beam [48, 57]. To maintain simplicity and generality of the analysis, independent of the type of load signal, we take only the static *PI* as the upper bound, but add the dynamical constraint mentioned before, stating that that the beam will “hit” the electrode if too much energy is introduced. To find it, we substitute $q = 1, \, \dot{q} = 0$ in the phase plane equation, Eq. (17), to isolate the initial conditions, geometry, and prestress that will bring about such a response, to produce the dynamic upper bound

26 $$\begin{aligned}  &   \dot{q}_0^2 + \omega _0^2 \left( \left( 1- \frac{P}{P_E} + \frac{\alpha s_{11}}{2 P_E} \left( 1 + q_0^2 - 2h_0^2 \right) \right) \right. \nonumber \\  &   \quad \left. \left( 1 + q_0 \right) - 2 h_0 \right) {{\left( q - 1 \right) }} = 0 \end{aligned}$$

implicitly connecting all parameters. Note that for $P/P_E = 0$, the upper bound is reduced to its stress-free version [46].

**Fig. 11:**
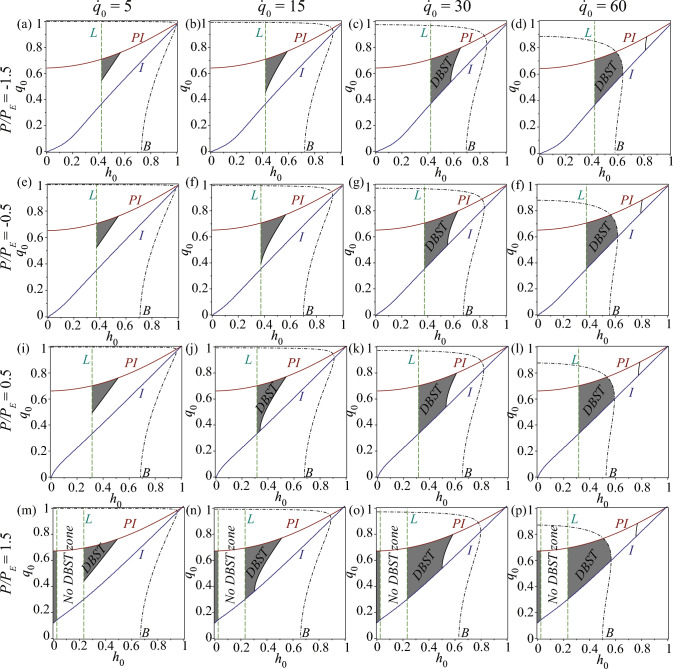
Condition for electrostatic dynamic bow snap-through (*DBST*) in terms of initial location ($q_0$) for d=0.15, and under different combinations of prestress and initial velocity. Solid blue, black and red lines correspond to initial location (*I*), the *DBST* necessary condition, and the pull-in (*PI*) curve, respectively, while the dashed green and dashed-dotted lines represent the latching condition *L* and the dynamic upper bound *B*. The dark grey areas represent where the conditions are met

Superimposing the condition from Eq. (19), the *PI* curve ($q_{PI}$), the dynamic upper bound (marked as $B = B \left( h_0, \, d, \, P/P_E \right)$) from Eq. (26), the latching thresholds ($h_{cr}$ & $h_{cr}^{\left( 2 \right) }$), and the initial location $q_I = q_{I} \left( h_0, \, d, \, P/P_E \right)$ on the $\{ h_0, \, q_0 \}$ plane for a constant d=0.15 and different $\{ P/P_E, \dot{q}_0 \}$ combinations, will result in the phase maps in Fig. 11. The agglomeration of all five curves and their interactions shows how the *DBST* area is limited by the two additional curves, namely the $q_{PI}$, and the upper bound *B*, represented by the red and black dashed-dotted lines, respectively. Note that the upper bound shifts to lower $h_0$ and $q_0$, as $\dot{q}_0$ increases. Per the case study from Sect. 3, it is possible to observe that with rising $\dot{q}_0$, the *DBST* area will also increase. However, that is not without limit, since the upper bound will start to reduce the allowable area for *DBST*. In other words, introducing an initial velocity will add more possibilities, but increasing it too much will diminish them under electrostatic load, as was postulated in the case study. Also note that the actuation maps, Figs. 3, 5, essentially constitute two different threads in the phase maps, Fig. 11, since the maps show all possible beams for the same *d*, while the case study shows the behaviour of a single beam. If one is to track a single line in the phase maps, then Fig. 11 shows that it will indeed increase with rising $\dot{q}_0$ but will then shrink in the presence of the upper bound. Recall that this change is in addition to the shift in $q_{PI}$ and $q_I$ with rising $P/P_E$. The former will attain lower values, while the latter will increase in value.

**Fig. 12:**
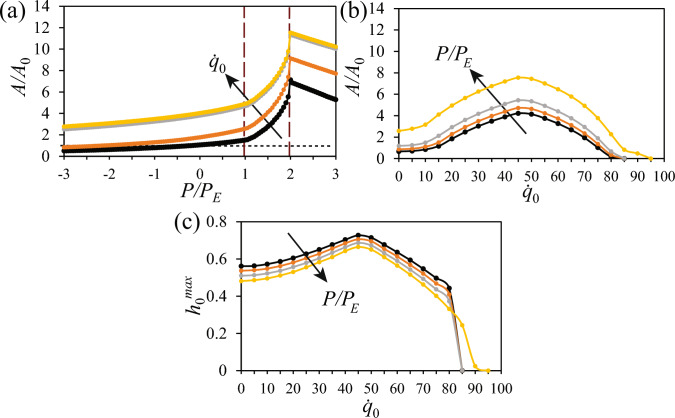
Condition area ratio ($A/A_0$) for d=0.15, against the area of a stationary and stress-free curved beam (i.e., $A_0$ is *A* for $P/P_E = 0$ and $\dot{q}_0 = 0$), corresponding to Fig. 11, for **a** $\dot{q}_0 = 5, \, 15, \, 30, \, 60$ in blue, black, orange, grey and yellow, respectively, as a function of $P/P_E$; and for **b** constant $P/P_E = -1.5, \, -0.5, \, 0.5, \, 1.5$ in black, orange, blue, grey, and yellow, respectively. The dotted horizontal black line represents $A/A_0 = 1$. Vertical dotted and dashed red lines represent $P/P_E = 0$ and $P/P_E = 1, \, 2$, respectively. **c** The maximum permissible initial elevation $h_0^{max}$ as a function of $\dot{q}_0$ for different $P/P_E$ (colour coding is identical to (**b**))

To better ascertain the behaviour of the *DBST* area in Fig. 11, we calculate the area (with respect to stress-free beam $P/P_E = 0$, and statically preloaded, $\dot{q}_0 = 0$) as a function of $P/P_E$ for four constant $\dot{q}_0$ (i.e., $A/A_0 = A/A_0 \left( P/P_E \right)$) and as a function of $\dot{q}_0$ for four constant $P/P_E$ (i.e., $A/A_0 = A/A_0 \left( \dot{q}_0 \right)$). At the current stage, the area can still be calculated via integration of numerically extracted fits. However, since this is a precursor to volume calculation and due to the multitude of curves and possible coincidences between them, we calculated the area by creating a grid composed of finite rectangular elements with an element size ϵ. To find the minimum required ϵ, we started with a convergence study, changing ϵ from ϵ=0.1 to $\epsilon = 7.8125 \times 10^{-4}$ to find that an element with $\epsilon = 1.5625 \times 10^{-3}$ can attain an error of about or lower than 1% when compared to the finest mesh, for four different cases, namely $\dot{q}_0 = 0, \, P/P_E = -1.5$; $\dot{q}_0 = 30, \, P/P_E = -1.5$; $\dot{q}_0 = 5, \, P/P_E = -1.5$, and $\dot{q}_0 = 60, \, P/P_E = 1.5$, encompassing the four dominant area permutations. Namely, when it crosses the latching threshold, when it crosses $q_I$, when there is a second area at low $h_0/d$, and when the upper bound *B* interacts with the condition, respectively. The final result is given in Fig. 12, showing the evolution of the area according to the prestress and as a function of the initial velocity, corresponding to Fig. 11. From Fig. 12a, b, we can see that while the area can increase in size as $P/P_E$ rises, it will do so with different trends and maximum values, depending on $\dot{q}_0$. The higher $\dot{q}_0$, so does the area. However, as was pointed out, increasing $\dot{q}_0$ too much will cause the boundary to overtake it, especially as $P/P_E$ gets higher. Note that $P/P_E = 1.5$ (the yellow curve), still maintains some area after all other curves have diminished to nought. This is attributed to the second area, which prompts when $h_{cr}^{\left( 2 \right) }$ materialises, which is overtaken at even higher $\dot{q}_0$. Note the nonlinear change when the trend changes at $P/P_E = 2$, attributed to the rising $q_I$ and decreasing $q_{PI}$. A trend that seemed linear in the mechanical counterpart, since it does not have an upper bound. In addition to area calculation, we also present the maximum initial elevation ($h_0^{max}$), present in each phase map, showing how it rises as $\dot{q}_0$ is increased and then lowered as the area starts to diminish. Once again, $P/P_E = 1.5$ can maintain a maximum elevation while all others have diminished, since it permits the existence of a second area of possibilities at low $h_0$, until the upper bound overtakes that too.

**Fig. 13:**
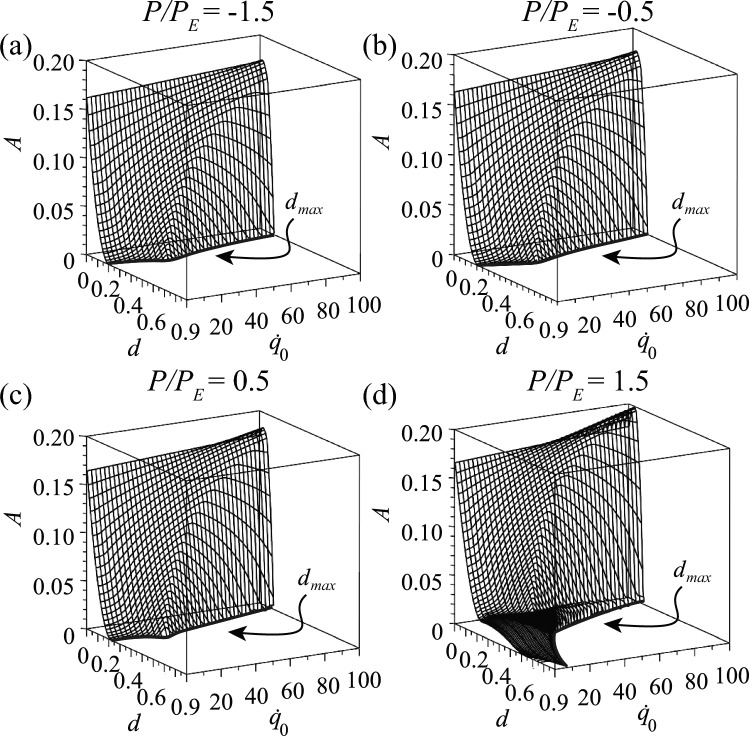
**a**–**d** Condition area, for four different $P/P_E$ values, corresponding to Fig. 12, showing how the area changes as a function of both *d* and $\dot{q}_0$

The electrostatic load has effectively introduced an additional parameter that was not present in the mechanical one, namely $g_0$. Therefore, if the mechanical condition rests in a four-dimensional space, then its electrostatic counterpart will reside in a five-dimensional space, a penteract. One way to represent it is by projecting the condition to a three-dimensional space, set out in $\{ h_0, \, d, \, q_0 \}$. However, due to the agglomeration of surfaces, depicting it would be difficult to understand. Instead, we span the curves from Fig. 12a, b to show how *A* changes as a function of both *d* and $\dot{q}_0$ for the four $P/P_E$ values, corresponding to the four $P/P_E$ values from Figs. 11, 12. The result is in Fig. 13, showing how the area decreases as *d* rises, until it is reduced to A=0. This trend was encountered in [47] as well, where it was shown that rising thickness-to-gap ratio will cause the *BST* threshold to shift upwards towards the *PI* curve, all while the *PI* curve changes as well. More specifically, when the prestress is negative, the *PI* curve will decrease with rising *d*, prompting a sharp decrease in area. As the prestress increases, the $d_{max}$ boundary is pushed to higher *d* since the *PI* curve begins to rise a bit, while the condition threshold increases to higher $q_0$. At $P/P_E = 1.5$, we see the highest $d_{max}$ in these cases due to the presence of a second latching threshold at low $h_0$, a feat that cannot come to pass for $P/P_E < 1$, since there is no $h_{cr}^{\left( 2 \right) }$ (and no second area). As for the initial velocity, we see that the area peaks at a certain $\dot{q}_0$, as expected, due to the presence of the dynamic upper bound, from which the area decreases. Observing the boundary of the surfaces, when A=0, reveals the upper bound for the maximum thickness-to-ratio, $d_{max}$, in each scenario, showing how it increases with rising $P/P_E$.

**Fig. 14:**
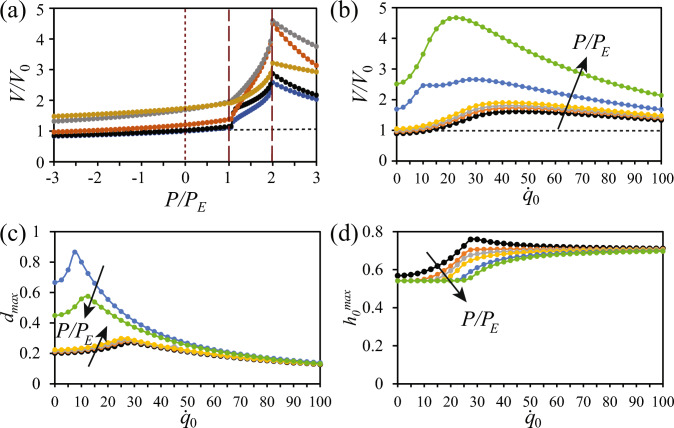
**a** The volume *V* of the dynamic electrostatic bow snap-through condition for different initial velocity, $\dot{q}_0$, as a function of the prestress, $P/P_E$, with respect to the volume of a stress-free curved beam (i.e., $V_0$ is *V* for $P/P_E = 0$). The dotted horizontal black line represents $V/V_0 = 1$. Vertical dotted and dashed red lines represent $P/P_E = 0$ and $P/P_E = 1, \, 2$, respectively. Blue, black, orange, grey and gold curves represent $\dot{q}_0 = 0, \, 5, \, 15, \, 30, \, 60$, respectively; **b** The volume *V* of the snap-through condition with respect to the volume of a stress-free curved beam, **c** the maximum allowable thickness, $d_{max}$, corresponding to the boundaries in Fig. 13 and **d** the maximum initial elevation, $h_0^{max}$, for different prestress values, $P/P_E$, as a function of the initial velocity, $\dot{q}_0$. Black, orange, grey, gold, blue, and green curves represent $P/P_E = -1.5, \, -0.5, \, 0, \, 0.5, \, 1.5, \, 2.1$, respectively

To find the volume for constant $P/P_E$ and $\dot{q}_0$, it is possible to sum the contributions of all points along each curve in Fig. 13. Once again, to attain a measure of the volume with good fidelity, another convergence study was conducted on the volume to find the required thickness increment, Δd. The study showed that a thickness of $\Delta d = 1.5625 \times 10^{-3}$ can attain an error of about or less than 1%. Conducting the calculation for constant $\dot{q}_0 = 0, \, 5, \, 15, \, 30, \, 60$ and constant $P/P_E = -1.5, \, -0.5, \, 0, \, 0.5, \, 1.5, \, 2.1$, will result in the curves in Fig. 14a, b, respectively. The first of the two shows how the volume is increased almost in a linear fashion until $P/P_E = 1$, from which all curves start to rise non-linearly until $P/P_E = 2$. After crossing $P/P_E = 2$, all volumes diminish in size, similar to what was seen in the stress-free case [47], albeit in different trends, depending on $\dot{q}_0$. It also shows that the rise between the curves is not injective either, since the volume rises with increasing $\dot{q}_0$ across $\dot{q}_0 = 0, \, 5, \, 15, \, 30$ shown as the blue, black, orange and grey curves, respectively, but will then diminish, as seen by $\dot{q}_0 = 60$ in gold. This is in line with previous observations, where it was seen that providing too much kinetic energy will hamper *DBST*. Such a trend is visible in Fig. 14b, showing the orthogonal plane to Fig. 14a, with constant $P/P_E$. From this perspective, it is visible that the curves are set higher as $P/P_E$ increases. Extracting the upper bound for $d_{max}$ and for $h_0^{max}$ shows that as the prestress rises, so does $d_{max}$, until $P/P_E = 2$, from which the overall $d_{max}$ will start to decrease. When it comes to $h_0^{max}$, it is decreased as $P/P_E$ is increased.

## Summary and conclusions

The paper has brought forth a unified necessary condition for dynamic bow snap-through (*DBST*), as well as upper bounds for electrostatically actuated bistable and latched beams while in the presence of prestress. Unlike the actuation scenario in [47], which dealt with a static preloading to a fixed point along the beam electrostatic equilibrium to gain enough strain energy, the loading scenario considered here is dynamic, and unlike the conditions set out in [46], prestress is taken into account to form a unified necessary condition, making the previous conditions private cases.

The precursor to this study, which focused on statically loaded beams to attain bow snap-through (*BST*), saw that it is possible to tune a given beam and enlarge the permissible space of possibilities for *BST* in terms of beam geometry (i.e., initial elevation-to-gap and thickness-to-gap ratios), and initial condition (which corresponds to voltage and is therefore interchangeable) thereby making it more feasible. However, it was also seen that there is an upper limit to this behaviour, since upon traversing $P/P_E = 2$, the pull-in and the post-prestress initial location start to limit the manoeuvring room of the beam within its electrostatic equilibrium curve, and as such, limit the number of options for prescribed $q_0$. As foretelling as the study was, limiting the actuation to static preloading has several disadvantages. First, the loading time is longer when compared to dynamic preloading, and secondly, dynamic preloading can introduce *BST* at lower voltages, much like dynamic snap-through can [48]. As such, introducing dynamic bow loading, witnessing its effect, and assessing its viability will go a long way in promoting this form of actuation and its application, making it a far more sustainable form of actuation compared to the classic one, where the electrode is pulling the beam from its convex side.

Before the derivation and extraction of a condition, the study introduced a case study, whereby two different forms of dynamic actuations are introduced, a finite pulse and a finite ramp, showing that it is indeed possible to attain *DBST* in a beam, depending on the maximum voltage of the signal, but also its duration, all while *DBST* can attain lower voltages when compared to static *BST*. Furthermore, it showed that as the prestress increases, the number of options will rise and then decrease, while in the latter of the two actuations, it was possible to compress the beam to a higher value. The leading hypothesis was that since the pulse signal inserts more kinetic energy, then too much energy may hamper *DBST*. Since the goal was to form a general condition, viable for all loading scenarios (including the static one), we therefore formulated a condition that depends solely on the initial conditions (i.e., $q_0, \, \dot{q}_0$), derived from a single degree-of-freedom (DoF) of a curved beam without the presence of damping, prompting a quartic and implicit equation that depends on the geometry of the beam, its prestress and the initial conditions. Upon demanding that the quartic equation has a vanishing discriminant to allow for the formation of a separatrix with three roots, a condition was achieved. An ensuing analysis was then conducted in two stages. First for a mechanical load, and then for an electrostatic one. The former has laid out the methodology and provided insights as to the behaviour of the new condition, set out in a four-dimensional space, showing how the volume of the condition, when projected unto $\{ h_0/d, \, \dot{q}_0/d, \, q_0/d \}$ will increase as a function of the prestress, until $P/P_E = 2$ is passed, from which the projection will decrease in volume. The electrostatic counterpart introduced a five-dimensional condition due to the additional dependence on the gap, a parameter that is irrelevant to the mechanical load, but paramount in the presence of a displacement-dependent one. The resulting condition, when projected to the space of $\{ h_0, \, \dot{q}_0, \, q_0 \}$, was shown to increase both as a function of prestress and as a function of the initial velocity, confirming our hypothesis from before, that inserting kinetic energy will increase the amount of options when compared to static preloading, but also that too much kinetic energy will hamper *DBST*. As before, compression seems to increase the number of options for *DBST* for all beams. The study ends with the extraction of the maximum elevation-to-gap ($h_0^{max}$), as well as maximum thickness-to-gap ($d_{max}$) ratios possible to attain *DBST*.

To conclude, the study has shown that dynamic *BST* can reduce the necessary voltage for snap-through by as much as $\approx \! 16 \%$, which can be added to the $\approx \! 54 \%$ when comparing it to a convex facing actuation [45]. The presence of a dynamic load can therefore facilitate bow actuation, making it more accessible and sustainable, as long as the amount of energy input is not too large, with prestress being used to tune a beam. Ultimately, the introduction of a dynamic load makes binary-based applications more viable, promoting efficient non-volatile and low-power bistable devices such as NVMMs, mechanical switches, threshold sensors, and other applications with lower voltages and faster transition times. The obtained necessary condition can be used as a guideline for engineers and researchers alike when constructing such devices, advancing the use and application of bistable structures in MEMS.

## Data Availability

The raw data is available via figshare in 10.6084/m9.figshare.31198033.
